# Micro-Computed Tomography Derived Anisotropy Detects Tumor Provoked Deviations in Bone in an Orthotopic Osteosarcoma Murine Model

**DOI:** 10.1371/journal.pone.0097381

**Published:** 2014-06-03

**Authors:** Heather A. Cole, Tetsuro Ohba, Jiro Ichikawa, Jeffry S. Nyman, Justin M. M. Cates, Hirotaka Haro, Herbert S. Schwartz, Jonathan G. Schoenecker

**Affiliations:** 1 Department of Orthopaedics and Rehabilitation, Vanderbilt University Medical Center, Nashville, Tennessee, United States of America; 2 Department of Pathology, Vanderbilt University Medical Center, Nashville, Tennessee, United States of America; 3 Department of Orthopaedic Surgery, Faculty of Medicine, University of Yamanashi, Yamanashi, Japan; University of Notre Dame, United States of America

## Abstract

Radiographic imaging plays a crucial role in the diagnosis of osteosarcoma. Currently, computed-tomography (CT) is used to measure tumor-induced osteolysis as a marker for tumor growth by monitoring the bone fractional volume. As most tumors primarily induce osteolysis, lower bone fractional volume has been found to correlate with tumor aggressiveness. However, osteosarcoma is an exception as it induces osteolysis and produces mineralized osteoid simultaneously. Given that competent bone is highly anisotropic (systematic variance in its architectural order renders its physical properties dependent on direction of load) and that tumor induced osteolysis and osteogenesis are structurally disorganized relative to competent bone, we hypothesized that μCT-derived measures of anisotropy could be used to qualitatively and quantitatively detect osteosarcoma provoked deviations in bone, both osteolysis and osteogenesis, *in vivo*. We tested this hypothesis in a murine model of osteosarcoma cells orthotopically injected into the tibia. We demonstrate that, in addition to bone fractional volume, μCT-derived measure of anisotropy is a complete and accurate method to monitor osteosarcoma-induced osteolysis. Additionally, we found that unlike bone fractional volume, anisotropy could also detect tumor-induced osteogenesis. These findings suggest that monitoring tumor-induced changes in the structural property isotropy of the invaded bone may represent a novel means of diagnosing primary and metastatic bone tumors.

## Introduction

Osteosarcoma characteristically manifests with rapid intra-osseous growth and extra-osseous extension in the metaphysis of long bones [1]. The typical biological characteristics of osteosarcoma are that of an osteolytic mass, often with areas of osteoid or cartilaginous matrix deposition, cortical thickening or destruction, and a periosteal reaction in areas of cortical transgression [2]–[4]. Radiographic imaging plays a crucial role in the diagnosis of osteosarcoma. The presence of aggressive radiologic features in a bone lesion is an indicator of its biological potential and mandates a tissue biopsy for histopathologic diagnosis.

Current methods for analyzing primary tumor growth in animal models of the disease are based on similar principles as the clinical diagnosis of osteosarcoma. Thus, plain radiographic analysis and histopathologic examination are the preferred methods for assessment of primary tumor growth, as these are generally simple, cost-effective techniques which provide a global characterization of the tumor. However, these methods are limited in detecting and quantifying the subtle biological characteristics of osteosarcoma such as periosteal reaction and osteolysis. An additional imaging modality routinely used in clinical diagnosis of osteosarcoma is computed tomography (CT), since it provides a spatial resolution of bony tissue that is sensitive to biological changes in bone structure [5] [6].

Currently, bone fractional volume of the metaphyseal region as measured by μCT is commonly used to assess tumor-induced osteolysis as a marker for local tumor growth of bone metastasis in animal models of prostate [7] and breast cancer [8]. As most tumors primarily induce osteolysis in these models, alterations in bone fractional volume correlate with tumor aggressiveness. However, osteosarcoma is more difficult to evaluate than most metastatic tumors because it typically presents with mixed osteolytic and osteoblastic components. As an expanding osteosarcoma alters the native bone architecture, our overarching hypothesis is that additional CT-derived parameters can accurately detect tumor growth and progression.

Competent bone is highly anisotropic; the systematic variance in its architectural order renders its physical properties dependent on direction of load [9]. Relative to competent bone, osteosarcoma-induced osteolysis and osteogenesis are structurally disorganized. Therefore we hypothesized that μCT-derived measures of anisotropy could be used to qualitatively and quantitatively detect osteosarcoma provoked deviations in bone, both osteolysis and osteogenesis, *in vivo*. To test this hypothesis we employed the murine intra-tibial model of osteosarcoma [5] [6].

## Materials and Methods

### Animal Model

Handling and care of all animals followed a protocol approved by the Institutional Animal Care and Use Committee at Vanderbilt University. Balb/C mice (Jackson Laboratories, Bar Harbor, ME) were housed at 22°C–24°C with a 12-hour light/dark cycle with standard mouse chow and water provided *ad libitum*. Mice are euthanized via CO2 overdose. To assess osteosarcoma growth *in vivo*, 6-week-old male BALB/c mice (N = 30) were injected with single-cell suspensions (1×10^5^) of an aggressive murine osteosarcoma cell line (K7M3) obtained from Dr. Kleinermann (MD Anderson Cancer Center, Dallas, TX). Cells were extracted by trypsin in PBS and kept on the ice. Mice were anesthetized and 10 µl of cell suspension was inserted into the proximal end of the tibia via a 30G½ needle as previously described [10] [11]. To confirm intra-tibial placement of the needle, model 1000 X-Ray Fluoroscopy obtained from Xi-Scan (Liverpool, UK) was employed before injection of cell suspension. Animals were evaluated for tumor burden by X-ray weekly. Mice were sacrificed after 3 (n = 5), 4 (n = 10) or 5 (n = 15) weeks (9, 10 or 11 weeks of age) by CO_2_ inhalation. To ensure complete collection of the left tibia and fibula, the distal femur and proximal talus were included in the dissected samples, as well as the surrounding soft tissues. Tissue was placed in 10% formalin for 48 hours and then stored in 70% ethanol.

### X-ray

A cabinet X-Ray device was from Faxitron (LX-60, Lincolnshire, IL). Digital X-ray images of the mice were collected for 8 sec at 35 kV. To facilitate congruency between radiographs, the anesthetized mice were placed in the prone position with hips in abduction causing external rotation of the tibia creating a lateral position. X-rays were taken once a week. The soft tissue area surrounding the tibia was established using the “polygon selections” tool from Image-J Version 1.44u (National Institute of Health). A circumference was drawn around the soft tissue of the tibia in the following manner: defining the circumference with borders defined by the patella superiorly, the tissue/tibia interface inferiorly, the skin anteriorly and the gastrocnemius muscle definition posteriorly. The area of the resultant circle was analyzed [12] [13].Tumor growth was calculated weekly as the percentage increase in size of the injected tibia compared with the paired contralateral, non-injected tibia.

### Micro-computed tomography (μCT)

#### Defining tibial segments

An ex vivo micro-computed tomography scanner was obtained from Scanco Medical AG (μCT40, Bruttisellen, Switzerland). Using a μCT40, 207 axial slices for two regions of interest (metaphysis and diaphysis) of equal size (2.48 mm in length per region) were acquired from each tibia at a 12 µm isotropic voxel size. For all scans, the energy settings were 70 kVp and 114 µA acquiring 1000 projections per 360° rotation with an integration time of 300 ms. For trabecular bone fractional volume (BV/TV) and trabecular non-metric indices such as connectivity density (Conn Dens) measurements, cortical bone was excluded from analysis to include only the trabeculae. For anisotropic measurement, cortical and trabecular bone were included. The diaphyseal region used for analysis was established as a region 6 to 8 mm distal from the metaphyseal zone (established as previously described utilizing the growth plate as a point of reference [15]). Each segment size (metaphyseal and diaphyseal) was 2.48 mm in length.

#### Analyses of defined tibial segments

Extent of osteolysis was determined by BV/TV and Conn Dens within the tibial metaphysis via contiguous cross-sections of the metaphyseal region. Mineralized tissue was distinguished from soft tissue with a threshold of 439.0 mgHA/cm^3^ and using a Gaussian noise filter (Sigma = 0.2 and Support = 1). One of the quantitative measures provided by the Scanco analysis of μCT images of bone is the degree of anisotropy (DA). Anisotropy is defined as a material's directional dependence of a physical property [14]. Here, structural anisotropy (i.e., directionality) of the bony elements was calculated using the mean intercept length (MIL) approach such that DA is the ratio of the length of the longest MIL to the length of the shortest MIL (this second order tensor is depicted graphically as an ellipsoid) [15]. Instead of using traditional test lines to determine MILs [16], the Scanco algorithm computes the distribution of MILs from surface projections [17]. Specifically, trabecular and cortical bone surfaces are triangulated [18] so that the area of the triangles can be projected onto a directional surface distribution (i.e., a vector normal to the surface for each triangle). This directional distribution is the inverse of the directional MILs [19]. Adapted for irregular surfaces, the surface projection-derived MIL distribution, and thus DA, can be determined for the metaphysis or diaphysis. In effect, highly organized bone structure correlates with higher anisotropic values, while disorganized, haphazard bone deposition is associated with decreased anisotropy (isotropy would by depicted graphically as a sphere). Anisotropy of bone within the metaphysis and within the diaphysis was used to determine the destructive capabilities of the tumor.

### Histology

For mice sacrificed at 10 weeks of age (4 weeks after tumor injection), dissected tibias were decalcified in 10% ethylene-diaminetetracetic acid (EDTA) for 7 days. All tissue was then processed into paraffin and stained with H&E and Martius scarlet blue as previously described [12].

### Statistics

The Mann-Whitney t-test was used to compare differences in BV/TV and anisotropy between control bone (no tumor) and bone injected with osteosarcoma cells. To compare the extent of tumor expansion into adjacent soft tissues over time, a Wilcoxon signed-rank test was used followed by Dunn's multiple comparison test for each time point. Correlations of endpoint X-ray measurements to BV/TV and anisotropy values were determined by Pearson's correlation coefficient. All tests were two-sided and *P*<0.05 was considered significant.

## Results

**Figure 1:**
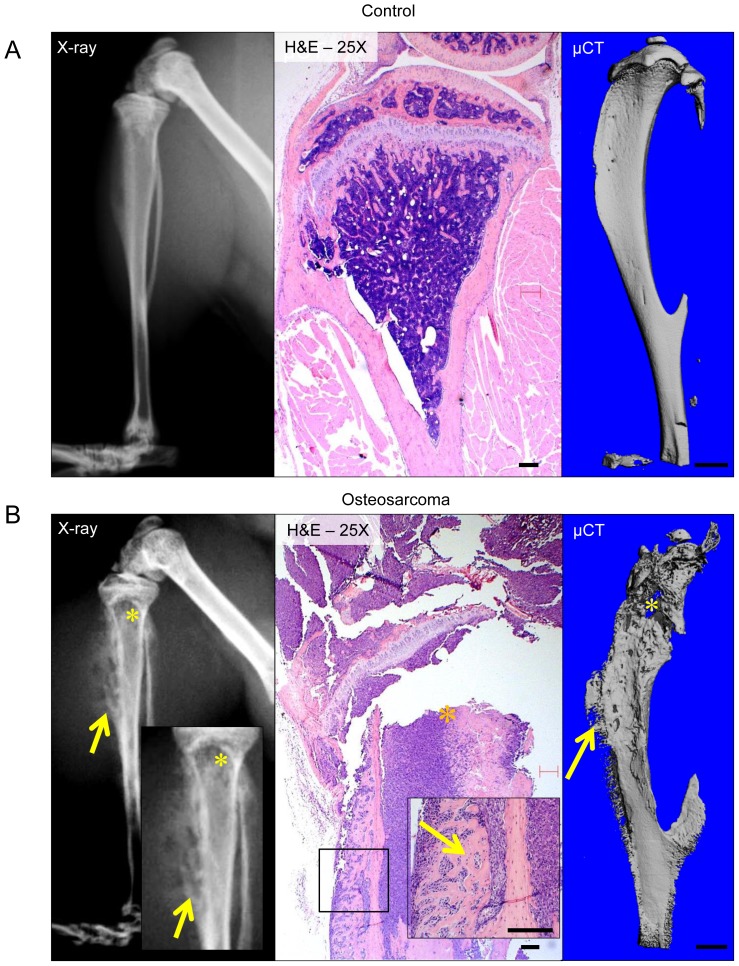
Imaging of primary osteosarcoma. Representative plain radiographs, histopathologic images, and 3D reconstructions from micro-computed tomography (μCT) of contralateral tumor free tibias. (A) and tibias injected with osteosarcoma cells (B). In tibias injected with osteosarcoma cells, trabecular bone in the medullary cavity is completely replaced by tumor, resulting in the osteolysis seen on radiographs (denoted by *). As tumor cells permeate and destroy cortical bone as they extend into the extra-osseous soft tissues, a vigorous osteoblastic periosteal reaction occurs, which corresponds to reactive woven bone deposition on the periosteal surface (arrows). Scale bar indicates 1.0 mm.

**Figure 2:**
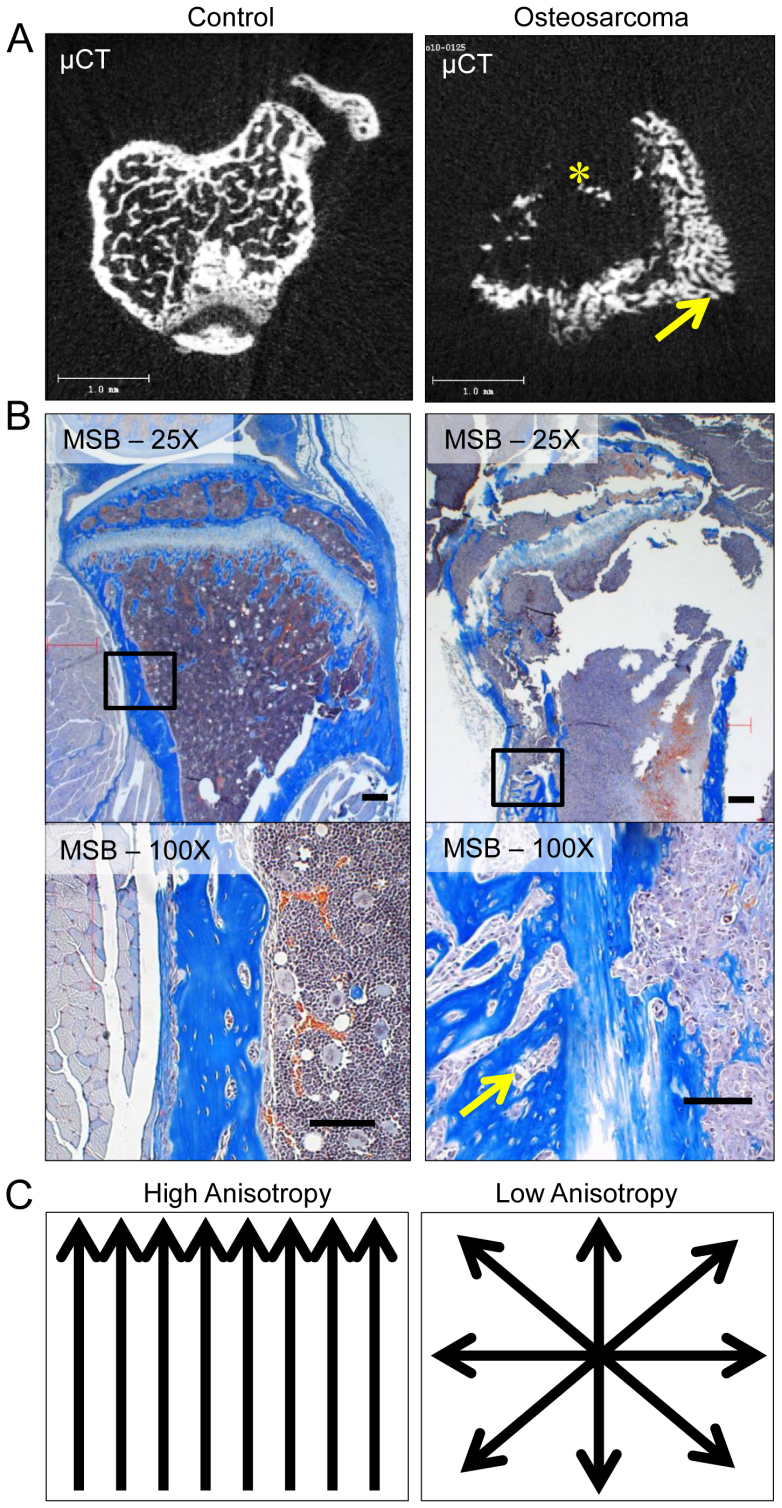
Degree of anisotropy measured by μCT as a means to quantify intra-osseous osteosarcoma. Representative examples of micro-computed tomography (μCT) (A) and histopathology (B) of control-injected and osteosarcoma-injected tibias is demonstrated. μCT analysis demonstrates marked osteolytic destruction of trabecular and cortical bone (indicated by *) and an associated periosteal reaction (yellow arrow) in the osteosarcoma-injected tibia. Martius scarlet blue-stained (MSB) sections (B) disclose the histologic correlates of the radiologic findings in osteosarcoma-injected tibias. Tumor cells replace the medullary cavity and permeate through native cortical bone (black arrow) resulting in pronounced osteolysis, as well as inciting a vigorous periosteal osteoblastic reaction (yellow arrow). The periosteal reaction results in a markedly disorganized pattern of bone deposition compared to normal bone. Anisotropy, a measure of bone organization demonstrated schematically in (C) might represent a novel parameter by which to quantify the local growth of primary osteosarcoma. Scale bar indicates 1.0 mm.

Four weeks after intra-tibial injection of osteosarcoma cells (at 10 weeks of age), radiographs, μCT and histopathologic analysis (Figures 1 and 2) demonstrate development of a lytic intra-osseous mass (Figure 1B) associated with extra-osseous extension. Extra-osseous tumor extension manifests as periosteal bone formation. In the medullary cavity, osteolytic areas where trabecular bone is completely replaced by tumor are readily apparent (Figures 1B and 2A). The pronounced periosteal reaction produced by extra-osseous tumor extension produces a markedly disorganized pattern of reactive woven bone deposition (Figure 2B). Based on these observations, we hypothesized that anisotropy; a measure of bone disorganization or directionality (demonstrated schematically in Figure 2C) may represent a novel method to quantify the growth of primary osteosarcoma *in vivo*.

**Figure 3:**
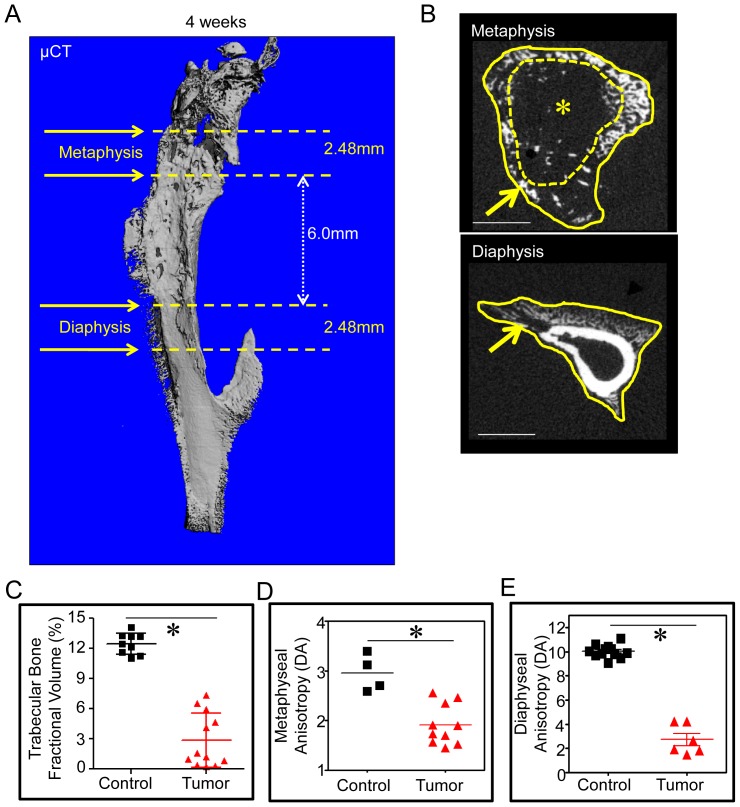
Defining areas for quantitative analysis of primary tumor growth by μCT. Areas where primary tumor growth parameters were measured (tibial metaphysis and diaphysis) are demonstrated in a 3D reconstructed μCT image (A). (Detailed in *Methods*.) (B) Representative 2D, axial images of the metaphysis and diaphysis demonstrate delineation of cortical bone between the endosteum (dotted yellow line) and periosteum (solid yellow line). The intramedullary or trabecular compartment is that surrounded by the endosteum. Areas of osteolysis (*) and periosteal osteoblastic reaction (arrow) are seen in both metaphyseal and diaphyseal regions of this osteosarcoma-injected tibia. Scale bar indicates 1.0 mm. (C) Relative tibia metaphyseal bone fractional volume (BV/TV) and (D) anisotropic measurements from tibial metaphyses and (E) diaphyses of control and osteosarcoma-injected mice demonstrate a significant degree of osteolysis (decreased BV/TV) of trabecular bone and decreased bone organization (manifested by lower anisotropic values) in osteosarcoma-injected tibias. *, P<0.01 compared to no tumor injection tibias.

As primary tumor growth was not limited to the metaphysis (Figure 1), two areas to be analyzed were defined. The method for measuring metaphyseal BV/TV has been previously described [15]. We defined the diaphyseal region of interest as that which lies 6.0 mm distal to the proximal tibial metaphysis (Figure 3A and B). Tibias injected with osteosarcoma cells show marked intramedullary osteolysis, as demonstrated by the significant decrease in metaphyseal BV/TV (control = 12.4±0.01 (n = 9), tumor side = 3.1±0.03 (n = 12), P<0.01) and Conn Dens (control = 219.3±61.7 (n = 9), tumor side = 49.3±45.9 (n = 12), P<0.01) (Figure 3C and Table 1). Areas of cortical destruction and periosteal bone formation in both the metaphysis (contralateral = 3.03±0.28 (n = 10), tumor side = 1.92±0.4 (n = 10), P<0.01) (Figure 3D and Table 1) and diaphysis (contralateral = 10.07±0.81 (n = 18), tumor side = 2.73±1.22 (n = 6), P<0.01) (Figure 3E and Table 1) show decreased anisotropy due to the loss of organized bone (cortex) and deposition of reactive woven bone associated with the periosteal reaction. These data suggest that both BV/TV and degree of anisotropy in the metaphysis and diaphysis are comprehensive parameters by which to assess growth of primary osteosarcoma.

**Table 1:** Bone microstructural properties of no or tumor-injected tibias were analaysied by uCT.

Time (weeks)	0	3	4	5
Metapyseal Anisotropy (DA)	3.03±0.28 (10)	2.3±0.37 (5)	1.92±0.4 (10)	1.94±0.39 (15)
Diaphseal Anisotropy (DA)	10.07±0.81 (18)	8.94±2.18 (5)	2.73±1.22 (6)	2.5±1.0 (15)
BD/TV (%)	12.4±1.08 (9)	11.0±0.74 (5)	3.1±0.27 (12)	3.0±0.26 (14)
Conn-dens (1/mm^3^)	219.3±61.7 (9)	188.2±62.1(5)	49.3±45.9 (12)	35.58±39.4 (14)

Mean±standard deviation (number of samples).

Time = weeks post tumor injection; BV/TV = bone volume fraction; Conn dens. = connectivity density.

**Figure 4:**
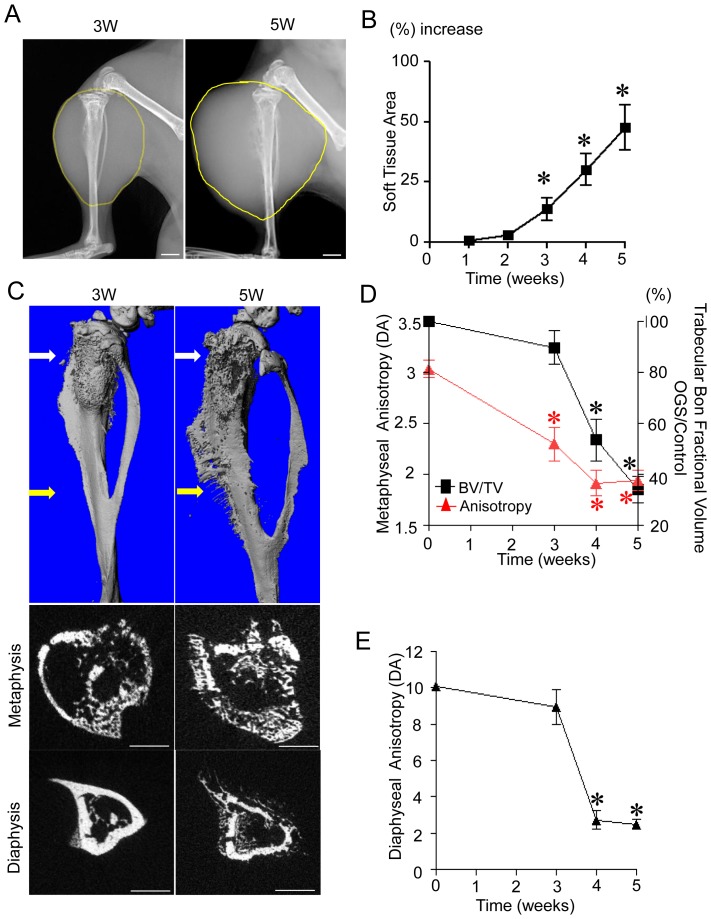
μCT analysis of extra-osseous extension of primary osteosarcoma. (A) Single-cell suspensions (1×10^5^) of murine osteosarcoma (K7M3) cells were injected into the tibia of mice (n = 30). Weekly radiographic evaluations were performed to monitor tumor growth. Representative X ray of tibias 3 and 5 weeks after tumor cell inoculation are shown. Scale bar indicates 1 mm. (B) Tumor growth was quantified by measuring the area define by soft tissue silhouettes in serial radiographs and recorded as the percent increase compared to the contralateral non-injected control tibia. *, P<0.01 compared to 1week post tumor injection group. (C) Representative 3D μCT images of osteosarcoma-injected tibias 3 or 5 weeks after injection with corresponding areas of measurement (metaphysis and diaphysis) are demonstrated. Scale bar indicates 1.0 mm. (D) Anisotropic values determined at the tibial metaphysis 3 (n = 5), 4 (n = 10) or 5 weeks (n = 15) after tumor injection (left side y-axis). Bone fraction volume of the tumor-laden tibial metaphysis determined by μCT normalized to the contralateral tumor free tibial metaphysis (right side y-axis indicates % relative decrease in bone fraction volume from 100%). (n = 5 at 3W; n = 12 at 4W; n = 14 at 5W) *, P<0.01 compared to no tumor injection tibias. (E) Anisotropic values determined at the tibial diaphysis 3 (n = 5), 4 (n = 10) or 5 weeks (n = 15) after tumor injection. *, P<0.01 compared to no tumor injection tibias.

To determine the efficacy of anisotropy by μCT analysis of primary osteosarcoma growth, we performed a time-course study using samples 3, 4 or 5 weeks post tumor injection. Plain radiographs illustrate tumor progression into the soft tissue of the tumor-bearing limb with significant expansion by 3 weeks (Figure 4A). Metaphyseal BV/TV measurements demonstrated significant osteolysis by 4 weeks after tumor injection (Figure 4C and D). In contrast, anisotropic values measured at the metaphyseal region showed significant changes as early as 3 weeks after tumor injection (3W post injection; contralateral = 3.03±0.28 (n = 10), tumor side = 2.3±0.37 (n = 5), P<0.01) (Figure 4C and 4D) whereas the diaphyseal region was significant at 4 weeks (Figure 4C and 4E).

To evaluate the influence of tibial needle injection itself, anisotropy values at the metaphyseal region and the diaphyseal region of tumor injected non-tumor developing tibia were compared to non-injected contralateral tibia. No significant difference between both groups indicated the tibial injection itself has not contributed to the change of bone anisotropy (Figure S1A and B). Finally, no significant Inter-observer error of anisotropy values measured at the metaphyseal region and the diaphyseal region by μCT was demonstrated (Figure S2A and B).

## Discussion

In this study, we show for the first time that μCT derived anisotropy detects tumor-provoked deviations in bone. We demonstrate that monitoring anisotropy allows for detection of destruction of native bone as a result of osteosarcoma growth as well as the exuberant periosteal reactive woven bone formation that accompanies cortical transgression and involvement of the extra-osseous compartment. We therefore conclude that monitoring anisotropy has the potential to be a superior method than the current gold-standard method of monitoring tumor induced changes in bone (BV/TV) in that it was found to be more sensitive in detecting bone destruction (osteolysis) and had the capacity to detect bone formation (osteogenesis).

Like trabecular bone architecture, the structural organization of cortical bone possesses a certain degree of anisotropy, as there is a primary axis (vertical in Figure 1A). Anisotropy is a fundamental property in the biomechanical behavior of bone that is a determinant of strength with respect to the loading mode (compression, tension, shear) [20]. Due to anisotropy, the structure of bone is divergent from the transverse to the longitudinal axes. In weight bearing long bones, as observed in this study, anisotropy in the longitudinal direction is favored. This gives the long bones high loading capacities and ability to compress; both of which are integral in withstanding body weight and upright movement. Through understanding the anisotropy of bone, various disease morphologies can be predicted by comparing anisotropic patterns by μCT analysis. Healthy bone has a high degree of anisotropy; whereas degraded bone or haphazard bone deposition has a lower degree of anisotropy [20]–[22].

Although osteosarcoma is a bone producing sarcoma, it is often associated with extensive osteolysis. Traditionally, BV/TV has been used to measure tumor-induced osteolysis [7] [23] [24]. In addition, anisotropy has been reported to reflect bone damage [25]. Recent studies have demonstrated quantitative heterogeneity of trabecular bone microstructure using anisotropy in osteoporosis patients [21], [22]. However, the efficacy of anisotropy determined by μCT analysis in measuring tumor-induced architectural changes is largely unknown. Multi-slice μCT reconstruction images distinguish the anatomic association between areas of bone destruction and adjacent periosteal reactions in sagittal, coronal, and axial planes. Although we demonstrate that the μCT parameters BV/TV and anisotropy can both be used to assess the degree of cortical and trabecular bone destruction and periosteal reaction, our data clearly show that in early tumor development (3weeks after injection), metaphyseal anisotropy was more sensitive than BV/TV. These findings allow earlier detection of tumor progression than the current method of BV/TV.

In addition to inducing bone destruction, osteosarcoma also induces bone production. As induction of periosteal reaction is commonly seen in osteosarcoma and other primary tumors of bone, it is used clinically as an indicator of aggressive potential. However, there are currently no methods to quantify the degree or extent of a periosteal reaction. We hypothesized that the extent of disorder induced in the bony architecture could be quantified by μCT analysis of anisotropy and thus provide a means to determine aggressive potential of primary bone tumors. Indeed, the degree of anisotropy measured at the tibial diaphysis involved by osteosarcoma clearly indicates increased disorder (low anisotropic values) of the bony architecture compared to controls. Thus, our findings indicate that μCT can accurately identify diaphyseal periosteal reactions and may be used to assess differences in regional tumor progression in this animal model. Anisotropy therefore represents a novel means of interrogating pathologic bone. To date, there have been no studies monitoring anisotropy in human osteosarcoma.

There are a few of limitations to this study. The osteosarcoma cell line used herein (K7M3) has a rapid growth rate and is not highly osteogenic. Thus, they produce a predominantly lytic lesion, as compared to more osteoblastic osteosarcomas [26]. As many osteosarcomas are mixed osteolytic and osteoblastic lesions, we cannot exclude the possibility that the bone fractional volume (BV/TV) and anisotropic values may be different when using other osteosarcoma cell lines. Second, the analysis of primary tumor growth was limited to two anatomic regions of the tibia to ensure practicality and cost efficiency of this methodology. Although areas of primary tumor were not included in the μCT analysis, the two regions defined for analysis appear to provide a reasonable assessment of primary tumor growth. Finally, in order to evaluate anisotropy in the metaphysis, we included both the intramedullary trabecular bone and cortical bone in the analysis. Ideally, trabecular bone would be analyzed separately from cortical bone. However, reliably distinguishing trabeculae from cortices in this region due to tumor-induced destruction of bone was difficult. Therefore, to eliminate a potential source of error, both bony compartments were included in the anisotropic analysis of metaphyseal bone.

In conclusion, this is the first paper to identify CT-derived measure of anisotropy, or tissue order, as a means of identifying tumor induced osteolysis and bone formation. Subsequent studies will be designed to assess if these preliminary animal model findings translate to the human condition: not only in osteosarcoma, but all tumors of bone. The potential impact of this work is that it provides a more sensitive detection system of bone cancer allowing for early and more comprehensive detection. In addition, if found true, its application would be rapid as it would only require an additional algorithm designed to calculate anisotropy to current CT systems without additional equipment.

## Supporting Information

Figure S1 **Compasion of negative controls.** Anisotropic measurements from (A) tibial metaphyses and (B) diaphysis of osteosarcoma-injected non tumor developing mice (n = 4) and non- injected contralateral tibia (n = 4). Non-tumor denotes osteosarcoma-injected non tumor developing mice, NS denotes no significant difference.
(TIFF)

Figure S2 **Inter-observer error of anisotropy values measured by μCT.** Anisotropic measurements from tibial metaphyses (A; n = 14) and diaphysis (B; n = 18) of non- injected control and osteosarcoma-injected mice by two independent observers. NS denotes no significant difference.
(TIFF)
