# Supplementary figures and images for: Micro-Computed Tomography Derived Anisotropy Detects Tumor Provoked Deviations in Bone in an Orthotopic Osteosarcoma Murine Model
Source: PLoS One. 2014 Jun 3;9(6):e97381. doi: 10.1371/journal.pone.0097381 (PMC4043681; doi:10.1371/journal.pone.0097381)

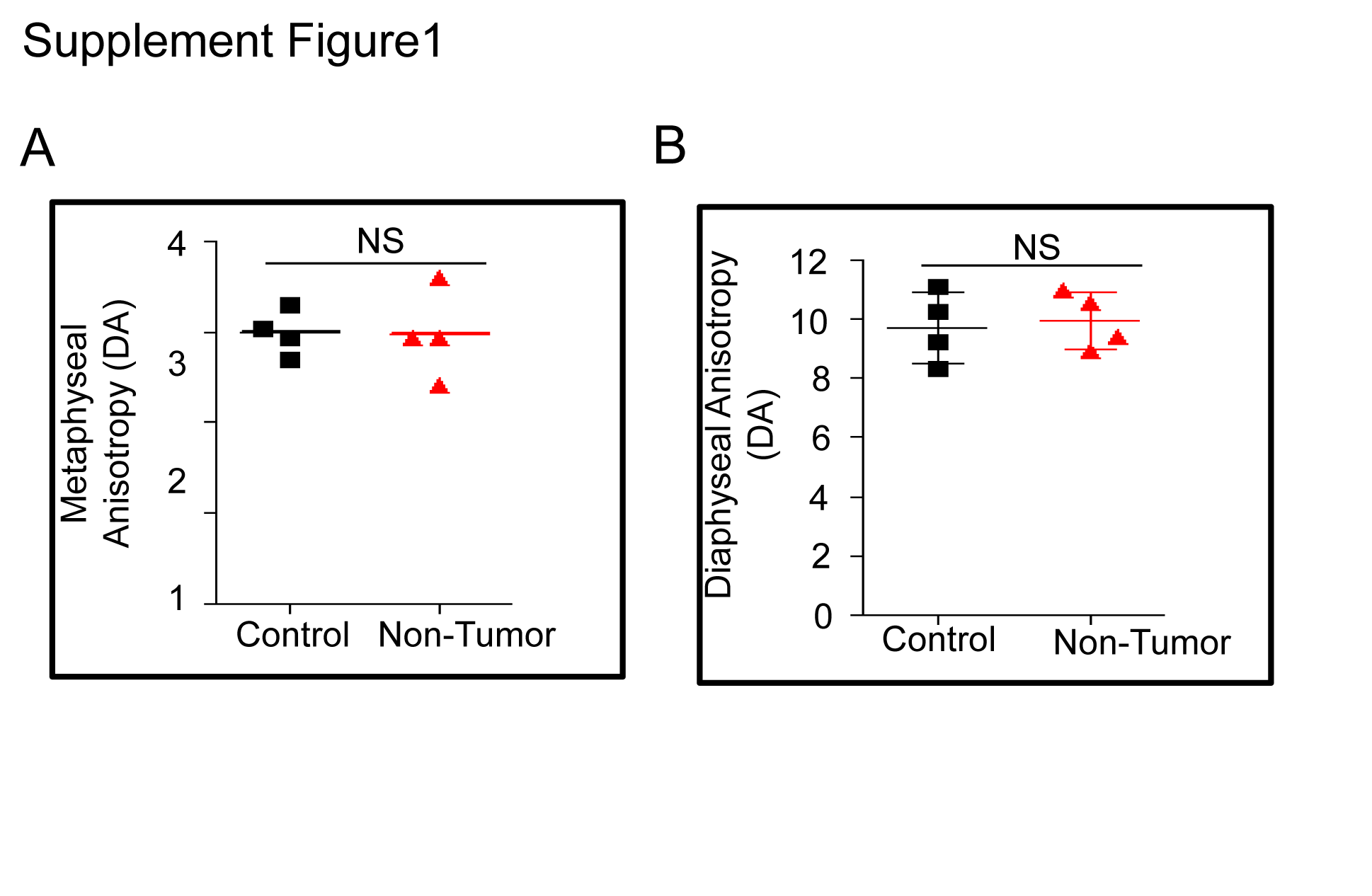

Supplement: Figure S1 — Compasion of negative controls. Anisotropic measurements from (A) tibial metaphyses and (B) diaphysis of osteosarcoma-injected non tumor developing mice (n = 4) and non- injected contralateral tibia (n = 4). Non-tumor denotes osteosarcoma-injected non tumor developing mice, NS denotes no significant difference. (TIFF) [file pone.0097381.s001.tiff]

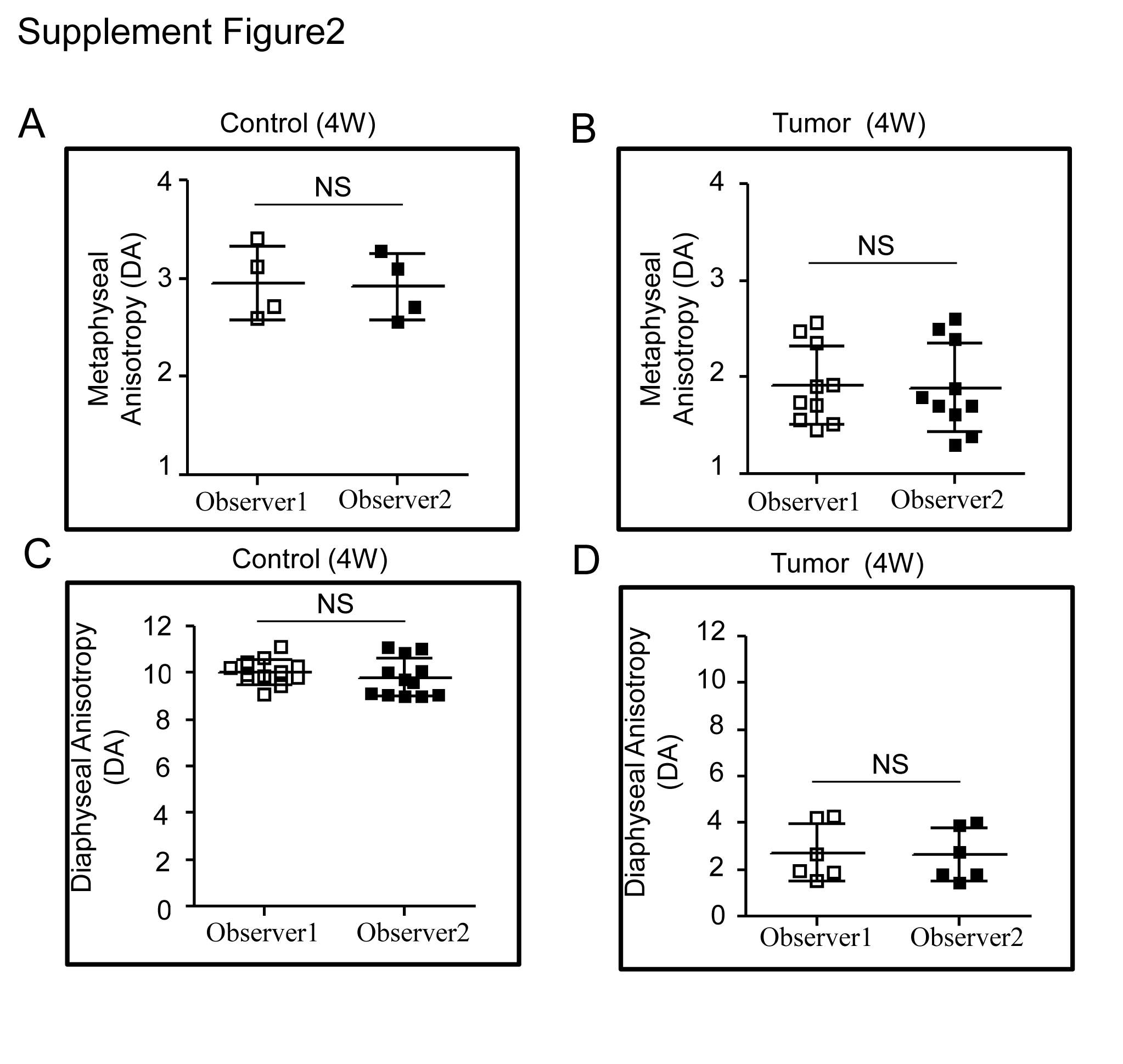

Supplement: Figure S2 — Inter-observer error of anisotropy values measured by μCT. Anisotropic measurements from tibial metaphyses (A; n = 14) and diaphysis (B; n = 18) of non- injected control and osteosarcoma-injected mice by two independent observers. NS denotes no significant difference. (TIFF) [file pone.0097381.s002.tiff]
